# Environmental Toxicity Assessment of Sodium Fluoride and Platinum-Derived Drugs Co-Exposure on Aquatic Organisms

**DOI:** 10.3390/toxics10050272

**Published:** 2022-05-23

**Authors:** Davide Di Paola, Fabiano Capparucci, Giovanni Lanteri, Rosalia Crupi, Ylenia Marino, Gianluca Antonio Franco, Salvatore Cuzzocrea, Nunziacarla Spanò, Enrico Gugliandolo, Alessio Filippo Peritore

**Affiliations:** 1 Department of Chemical, Biological, Pharmaceutical, and Environmental Science, University of Messina, 98166 Messina, Italy; dipaolad@unime.it (D.D.P.); fcapparucci@unime.it (F.C.); glanteri@unime.it (G.L.); mrnyln96c42f112s@studenti.unime.it (Y.M.); frnglc96h11f112o@studenti.unime.it (G.A.F.); aperitore@unime.it (A.F.P.); 2 Department of Veterinary Science, University of Messina, 98166 Messina, Italy; rcrupi@unime.it (R.C.); egugliandolo@unime.it (E.G.); 3 Department of Pharmacological and Physiological Science, Saint Louis University School of Medicine, St. Louis, MO 63104, USA; 4 Department of Biomedical and Dental Sciences and Morphofunctional Imaging, University of Messina, 98166 Messina, Italy

**Keywords:** fluoride, platinum-based antineoplastic drugs, zebrafish

## Abstract

Pharmaceuticals are widely acknowledged to be a threat to aquatic life. Over the last two decades, the steady use of biologically active chemicals for human health has been mirrored by a rise in the leaking of these chemicals into natural environments. The aim of this work was to detect the toxicity of sodium fluoride (NaF) exposure and platinum-derived drugs in an ecological setting on aquatic organism development. From 24 to 96 h post-fertilization, zebrafish embryos were treated to dosages of NaF 10 mg/L^−1^ + cisplatin (CDDP) 100 μM, one with NaF 10 mg/L^−1^ + carboplatin (CARP) 25 μM, one with NaF 10 mg/L^−1^ + CDDP 100 μM + CARP 25 μM. Fluoride exposure in combination with Cisplatin and Carboplatin (non-toxic concentration) had an effect on survival and hatching rate according to this study. Additionally, it significantly disturbed the antioxidant defense system and increased ROS in zebrafish larvae. NaF 10 mg/L^−1^ associated with CDDP 100 μM and CARP 25 μM, increased the production of apoptosis-related proteins (caspase 3, bax, and bcl-2) and the downregulation of acetylcholinesterase (AChE) activity, while no effect was seen for the single exposure.

## 1. Introduction

Fluoride is used in the production of fluoridated dental products as well as it is used in drinking water fluoridation processes [1,2]. The World Health Organization establishes an acceptable level for drinking water, which ranges from 0.7 to 1.0 mg/L^−1^. However, fluoride concentrations of up to 20 mg have been recorded in some countries [3]. Sodium fluoride NaF was reported to be present in water at a range of 9 mg L^−1^ to 17 mg L^−1^ [4] and concentrations between 1.5 mg L^−1^ and 7 mg L^−1^ varying from country to country [4,5]. Epidemiological studies reveal that populations living in areas with high fluoride levels are more susceptible to neurological or intellectual problems [6,7]. However, the toxic effects of high amounts of fluoride in the environment, do not only present a danger to humans, but also to the different species that inhabit the environments in which these substances accumulate [8]. Fluoride-related central nervous system (CNS) function has been shown to be sensitive in experimental tests [9,10,11]. Fluoride has been shown in experimental animals to produce neurotoxicity, including effects on learning and memory [12,13]. This chemical builds up in numerous parts of the brain, causing a variety of symptoms, including decreased synaptic cleft width [14]. According to available research, excessive fluoride can harm neurons and synapses by causing free radicals and lipid peroxidation, which may increase the vulnerability of neurons to excitotoxicity [15,16]. Reduced nicotinic acetylcholine (ACh) receptors and histological alterations in brain cells of rats with impaired learning and memory have been highlighted after fluoride exposure [11,17,18]. ACh is promptly cleaved into choline and acetate by acetylcholinesterase (AChE), which has been reported as a well-known biomarker for a variety of pollutants [19]. Zebrafish have emerged as an alternate perspective for understanding neurotoxicant chemicals in this context. Fluoride can pass the blood–brain barrier in zebrafish, causing detrimental effects on neural cells and eventually mental impairment [20,21] found that 72.12 mg/L^−1^ NaF changes the expression pattern of genes involved in inflammation, apoptosis, and DNA repair in zebrafish. Furthermore, NaF genotoxic and mutagenic potential has been attributed to it [22]. In the current study, we looked into whether fluoride-induced neurotoxicity was linked to oxidative stress changes under these experimental NaF exposure circumstances. The World Health Organization (WHO) lists anticancer drugs as one of the eight most often used types of medicine on the planet. In chemotherapy, platinum-based antineoplastic drugs are commonly utilized. They include cisplatin (CDDP), carboplatin (CARP), and oxaliplatin (OXA), which crosslink DNA strands or generate DNA-protein crosslinks in cancer cells [23,24]. Several studies have demonstrated that exposure to platinum or its derivatives, such as cisplatin, can cause genotoxic and teratogenic consequences in zebrafish embryonic development [25,26]. Several studies have found rising platinum group element concentrations in several areas of the water ecosystem, including drinking, ground, and surface waters [27]. The largest sources of platinum compounds in the environment are emissions from automotive catalytic converters and hospital effluents. A study on wastewater samples from cancer departments in hospitals reported high levels of carcinogenic platinum compounds including oxaliplatin, varying from 4.7 to 145 micrograms/liter [28]. There is a lot of ecotoxicological data on anticancer medications in the literature [29]. Unfortunately, there are limited data on acute antitumoral drug exposure as a pollutant in the environment and its effects on aquatic species, particularly the most vulnerable forms such as larvae [21]. The Danio rerio Fish Embryo Toxicity (FET) assay is a commonly used methodology for determining the toxicity of environmental pollutants [30,31]. Other consequences on development, such as morphological abnormalities, delayed development, pericardial edema, and yolk sac edema, can be shown in the FET test. This is a promising technology that proposes medicines (and other substances) and their toxicity processes could be studied using fish lines at lower concentrations because surface water contains minimal amounts of environmental contaminants. Furthermore, as a whole-animal drug screening platform, zebrafish can swiftly identify medicines with evident developmental toxicity or absorption difficulties [27]. Fluoride is ubiquitous in the environment and is always present in plants, soils, and phosphate fertilizers [32]. It has been shown that a large proportion of the total F in ash is apparently soluble in the digestive system of grazing animals [33]. Thus, even if at concentrations below the toxicity threshold, the interaction of NaF with other pollutants could give harmful effects. In particular, the overwhelming incidence of carcinomas treated with platinum derivative therapies leads to increased levels of these drugs at appreciable environmental levels. Therefore, the increased presence of anticancer drugs in the environment and the high presence of NaF could present a potential toxic crosslink for animal species living there and consequently for human health.

## 2. Materials and Methods

### 2.1. Selection of Concentrations

Considering the detected environmental concentrations of NaF with a very wide range, we performed preliminary analyses to determine the toxic concentrations on zebrafish embryonic development. For these reasons, we opted to use NaF concentrations ranging from 5 to 10 to 25 to 50 mg/L^−1^. In order to identify the suitable concentrations of CARP and CDDP (both pharmaceuticals were detected in different aquatic environments, with concentrations ranging from ng/L^−1^ up to mg/L^−1^) (0.687 for CARP and 0.250 for CDDP) [34], and time points established by OECD guidelines for the following experiments, CARP concentrations of 25, 50, 100 and 150 μM were added into embryo medium and were applied to observe the larvae morphology until 96 hpf. The same procedure used for CARP was also used to select CDDP concentrations. CDDP concentrations of 5, 10, 25, and 50 μM were added into embryo water and were applied to observe the larvae morphology until 96 hpf, as seen previously [35].

### 2.2. Solutions Preparation

CARP (Carboplatin TEVA) 600 mg/60 mL and CDDP (Cisplatin TEVA) 1 mg/mL were purchased from (Teva Italia S.r.l Via del Mulino, 1, 20,090 Assago (MI) Italy. The solutions were diluted in embryo medium (15 mM NaCl, 0.5 mM KCl, 1 mM CaCl_2_, 1 mM MgSO_4_, 0.15 mM KH_2_PO_4_, 0.05 mM Na_2_HPO_4_, 0.7 mM NaHCO_3_ at pH 7.3) obtaining concentrations ranging from 10, 25, 50 μM to 100 μM of CARP and 2,550,100 and 150 μM of CDDP, respectively. Solutions have been placed in 24 well plate (Labsolute, Th. Geyer GmbH & Co. KG, Dornierstr. 4–6 D-71272 Renningen, Germany.), NaF was purchased from Sigma Aldrich, St. Louis, MO, USA.

### 2.3. Zebrafish Maintenance and Breeding

For the generation of embryos, wild type (WT) adult zebrafish aged 6 months were employed. Zebrafish were raised in the Department of Veterinary Sciences, University of Messina, Italy, in the Centre for Experimental Fish Pathology (Centro di Ittiopatologia Sperimentale della Sicilia—CISS). Since 2006, CISS has been accredited for the use and development of aquatic models for research, and all procedures followed EU/63/2010 DL. The fish were fed dry and live food twice a day at a rate of 3% of their body weight (BW). Mature females and males were coupled in a 2:1 ratio for successful reproduction. The eggs were retrieved the next day, bleached, and non-fertilized eggs were discarded. According to Directive 2010/63/EU and relating Italian DL 26/2014 on the protection of animals used for scientific purposes, experiments on zebrafish larvae up to five days (120 h) post-fertilization and particularly ZFET are paired with alternative methods and thus they do not need ethical approval.

### 2.4. Zebrafish Embryo Toxicity (ZFET) Assay

The toxicity of our substances solutions was established following the OECD guideline (OECD, Test No. 236: fish embryo acute toxicity (FET) test). We have conducted some preliminary experiments using different concentrations of NaF (5, 10, 25, and 50 mg/L^−1^); CDDP (5, 10, 25, and 50 μM); CARP (25, 50, 100, and 150 μM) to find a minimal full non-toxic dose (20 eggs for each experimental group, three different experiments). After selecting the highest non-toxic concentrations from previous experiments (100 μM, 25 μM, and 10 mg/L^−1^ for CDDP, CARP, and NaF, respectively, we have proceeded as previously seen [36]. We used four experimental groups, which have been divided as follows: one with embryos were exposed to NaF 10 mg/L^−1^ + CDDP 100 μM, one with an exposure of NaF 10 mg/L^−1^ + CARP 25 μM, one with NaF 10 mg/L^−1^ + CDDP 100 μM + CARP 25 μM, plus a negative control group (we used 40 eggs for each experimental group, the experiment was repeated three times). At 24, 48, 72, and 96 h post-fertilization (hpf), the whole mortality and developmental anomalies of embryos and larvae were monitored and documented [24]. Furthermore, embryonic abnormalities were assessed as a teratogenic endpoint during development. The proportion of hatching and mortality were also calculated. Images and movies were captured using a stereomicroscope (Leica M205 C, Leica Microsystems Srl, Buccinasco Milano, Italy). Four different endpoints were evaluated every 24 h to see whether there were any abnormalities:

1. embryo coagulation—can also occur within a few hours of the start of exposure and indicates a generic acute toxic effect;
2. lack of somite formation— if a somite is not visible 12 h after fertilization, the embryo will not develop further, resulting in death;
3. non-detachment of the tail— the dissociation of the tail from the yolk sac can be seen 24 h after fertilization, indicating that the embryo is growing normally;
4. absence of heartbeat— the absence of a heartbeat 30 h after fertilization implies that the embryo has died; embryo coagulation and the absence of a heartbeat were utilized as endpoints of mortality.

### 2.5. Total RNA Extraction and RT-PCR

The total RNA from zebrafish larvae (20 randomly selected larvae from surviving 96 hpf larvae per experimental group of each experiment) was homogenized and isolated in 0.50 mL TRIzol reagent (Invitrogen, Waltham, MA, USA) according to the manufacturer’s instructions. The manufacturer’s instructions were followed to isolate total RNA. The quality of the RNA in each sample was determined using the ratio of absorption at 260–280 nm and the banding patterns on a 1 % agarose formaldehyde gel. Gel electrophoresis was used to assess RNA quality, and NanoDrop 2000 (Thermo Scientific, Waltham, MA, USA, iScript RT-PCR kit (Bio-Rad, Hercules, CA, USA) was used to determine concentration and to synthesize first-strand cDNA according to manufacturer’s recommendations. The reverse transcription master mix was prepared by adding to 1 μg of RNA template the iScript RT Su-permix (5× RT supermix with RNase H+ Moloney (gray cap, 25 or 100 reactions) murine leukemia virus (MMLV) reverse transcriptase, RNase inhibitor, dNTPs, oligo (dT), random primers, buffer, MgCl2, and stabilizers) and the nuclease-free water. The complete reaction mix was incubated in a thermal cycler (Priming 5 min at 25 °C, Reverse transcription 20 min at 46 °C, RT inactivation for one minute at 95 °C). Real-time PCR was performed with a 20-μL volume containing 10-μL of 1× SsoFast EvaGreen Supermix (Bio-Rad, Hercules, CA, USA), 1 μL of cDNA, 7 μL of RNase/DNase-free water, and 500 nM each primer. PCR conditions were initial denaturation at 95 °C for 15 min, followed by 45 cycles of amplification at 95 °C for 20 s and 60 °C for 40 s. The StepOnePlus Real-Time PCR System (Applied Biosystems, Foster City, CA, USA) was then used to execute a final extension at 60 °C for 60 s and a hold at 4 °C. The RT-PCR technique was adapted from a previous study [37]. Each gene in the present study was assessed in triplicate. The sequences of primers for the real-time PCR are shown in Table 1. β-actin was used as an internal control for normalizing relative expression levels between samples. Data analysis was performed using the 2^−∆∆Ct^ method, and the results are expressed as fold changes.

### 2.6. MDA, Antioxidant Enzyme and Acetylcholinesterase (AChE) Activity Measurement

Each plate’s larvae were defrosted and homogenized on ice with 1 mL of ice-cold physiological saline. By centrifuging the homogenate at 4000× *g* for 15 min at 4 °C, the supernatant was collected. As previously mentioned, the concentration of MDA, SOD, and CAT in the supernatant was determined using commercial kits (Nanjing Jiancheng Bioengineering Institute, Nanjing, China) [38,39]. Acetylcholinesterase (AChE) activity was determined as previously described [40]. The enzyme activities are expressed as specific enzyme activities in enzyme units (U) per mg of protein.

### 2.7. Data Analysis

Microsoft Excel was used to evaluate all of the raw spreadsheet data. GraphPad Prism 8.3.1 software (GraphPad, San Diego, CA, USA, 2020) was used to plot and statistically analyze graphs. To find significant differences between the mean values, a two-way ANOVA analysis of variance was utilized (ANOVA-SNK) (two variables such as time and different exposures). The data were tested for normal distribution with the Kolmogorov–Smirnov test (*p* < 0.05) and they were represented as mean ± standard error of the mean (SEM) (alpha value of 0.05).

## 3. Results

### 3.1. Viability and Morphology of Zebrafish Embryos after CDDP, CARP, and NaF Exposure

The CARP 150 μM group induced a rise in mortality, in the zebrafish embryos during the 96 h exposure. No hatched larvae were observed in the zebrafish exposed to CARP at a concentration of 150 μM, and this condition was observable in 100% of larvae exposed to 150 μM CARP concentration at 96 hpf (Table 2). No statistically significant toxicity was noted in the 100, 50, and 25 μM groups compared with the control group. Next, we analyzed different concentrations of CDDP for lethal end points during embryonic development. CDDP doses of 5, 10, 25, and 50 μM were added to embryo water and used to observe the larvae morphology until 96 hpf in order to determine the best concentrations and time points for the next experiments. Table 2 shows that CDDP doses of 5,10 and 25 μM had no effect on zebrafish morphology after 96 h when compared to the control group (Table 2). In zebrafish embryos, the CDDP 50 μM group caused an approximately 60% mortality and no hatched larvae at 96 hpf (Table 2). At the highest NaF concentration 50 mg/L^−1^ more than 80% of the embryos were deceased at 72 hpf and no embryos reached hatching. The NaF 25 mg/L^−1^ group showed a slight mortality rate and delay on hatching compared to the control group. The NaF 10 and 5 mg/L^−1^ showed no significant sign of developmental toxicity or delay on hatching at 96 hpf compared to the control group (Table 2).

### 3.2. Survival and Hatching Rate

**Figure 1:**
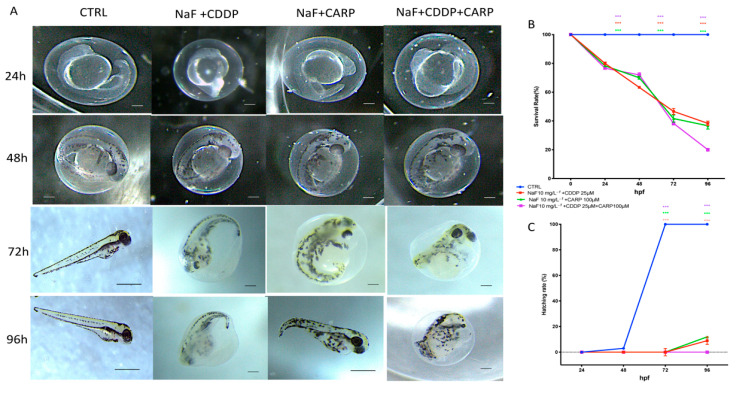
The morphological abnormalities in zebrafish caused by different NaF associations:NaF 10 mg/L^−1^ + CDDP 25 μM; NaF 10 mg/L^−1^ + CARP 100 μM and NaF 10 mg/L^−1^ + CDDP 25 μM + CARP 100 μM (**A**), survival rate (**B**), and hatching rate (**C**). Images were taken from the lateral view under a dissecting microscope (magnification 25). Scale bar, 500 mm.

Embryo cumulative mortality after exposure to NaF 10 mg/L^−1^ + CDDP 25 μM; NaF 10 mg/L^−1^ + CARP 100 μM and NaF 10 mg/L^−1^ + CDDP 25 μM + CARP 100 μM is reported in Figure 1A. Survival rate was documented at 24, 48, 72, and 96 hpf. Figure 1A shows that NaF 10 mg/L^−1^ + CDDP 25 μM + CARP 100 μM caused 80% death at 96 hpf, resulting in a massive mortality rate compared to the control group. Moreover, also in the other two exposure groups, NaF 10 mg/L^−1^ + CDDP 25 μM and NaF 10 mg/L^−1^ + CARP 100 μM, a high peak of mortality at 96 hpf was found, which showed that every combination of NaF 10 mg/L^−1^ with CDDP 25 μM/CARP 100 μM had an impact on zebrafish larvae development. As hatching is a critical time in zebrafish embryogenesis, the hatching rate is one of the most important indices for determining developmental toxicity in zebrafish. Embryos started hatching at 48 hpf and finished at 96 hpf, according to previous investigations. Our results showed that no exposure group embryos had hatched by 72 hpf, while in the group with the concentration of NaF 10 mg/L^−1^ + CDDP 25 μM + CARP 100 μM, no embryos still hatched at 96 hpf. Therefore, as shown in Figure 1C, the embryo hatching rate was reduced in all the exposure groups versus the control. 

### 3.3. Effect of NaF, CDDP, and CARP on Lipid Peroxidation and Stress Oxidative Pathway

**Figure 2:**
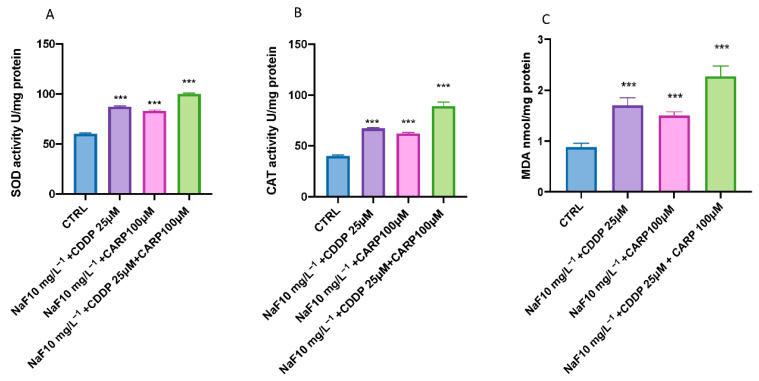
Effects of Naf 10 mg/L^−1^ + CDDP/NaF 10 mg/L^−1^ + CARP/NaF 10 mg/L^−1^ + CDDP + CARP exposure on activities of SOD (**A**), CAT (**B**) MDA (**C**), in the larval zebrafish. Embryonic zebrafish was exposed to these solutions from 24 to 96 hpf. Data are expressed as the mean ± SEM of three replicates (about 20 larvae per replicate). The asterisk denotes a statistically significant difference when compared with the CTRL: *** *p*< 0.0001 versus control.

The results showed an increase in MDA, SOD, and CAT activities related to oxidative damage after NaF 10 mg/L^−1^ + CDDP 25 μM/NaF 10 mg/L^−1^ + CARP 100μM exposure (Figure 2). In fact, co-exposure with NaF + CDDP and NaF + CARP at 96 h compared to the control group showed a significant increase of MDA levels (Figure 2C). Moreover, the full association group (NaF 10 mg/L^−1^ + CDDP 25 μM + CARP 100μM) did show a huge notable effect on oxidative stress in larval compared CTRL group and also an increase in MDA, SOD, and CAT activities versus the other two treated groups (Figure 2). 

### 3.4. Apoptosis Process

**Figure 3:**
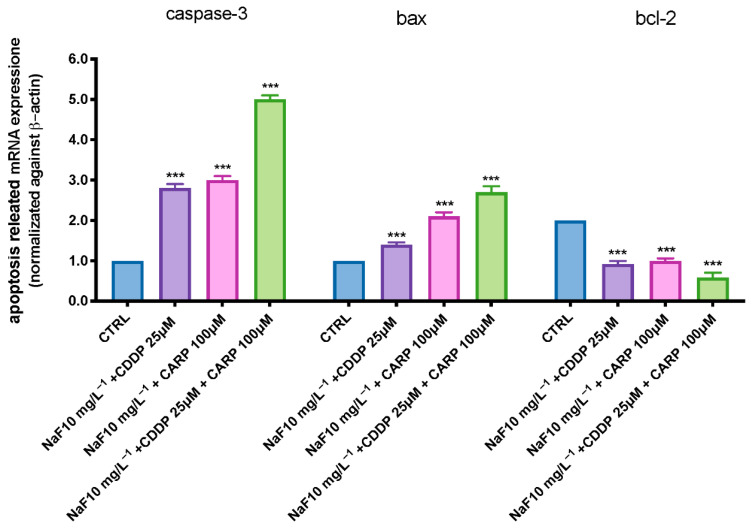
The NaF associations groups exposure effects on cell death zebrafish embryos. Related gene expression levels of apoptotic pathway in zebrafish embryos exposed to NaF 10 mg/L + CDDP 25 μM (A); NaF 10 mg/L^−1^ + CARP 100 μM (B); NaF 10 mg/L^−1^ + CDDP 25 μM + CARP 100 μM (C). The fold change from the CTRL group is considered to reflect the mRNA expression levels. *** *p* < 0.001 versus control.

We employed RT-PCR to assess the mRNA expression levels of larvae exposed to three exposition groups (NaF 10 mg/L + CDDP 25 μM; NaF 10 mg/L^−1^ + CARP 100 μM; NaF 10 mg/L^−1^ + CDDP 25 μM + CARP 100 μM) at 96 hpf. The expression levels of apoptosis-related genes (caspase-3 and bax) rose with NaF 10 mg/L^−1^ + CDDP 25 μM; NaF 10 mg/L^−1^ + CARP 100 μM exposure dose, according to the RT-PCR data. Contrary, increasing NaF 10 mg/L^−1^ + CDDP 25 μM; NaF 10 mg/L^−1^ + CARP 100 μM exposure levels, bcl-2 mRNA expression was downregulated (Figure 3). Moreover, when compared to the other two exposure groups, NaF 10 mg/L^−1^ + CDDP 25 μM + CARP 100 μM exposition dramatically increased caspase-3 and Bax levels expression (Figure 3) (*p* value < 0.0001). In the NaF 10 mg/L^−1^ + CDDP 25 μM + CARP 100 μM group, however, the anti-apoptotic protein bcl-2 expression was considerably reduced (*p* value < 0.0001). 

### 3.5. AChE Activity after NaF, CDDP, and CARP Exposure

**Figure 4:**
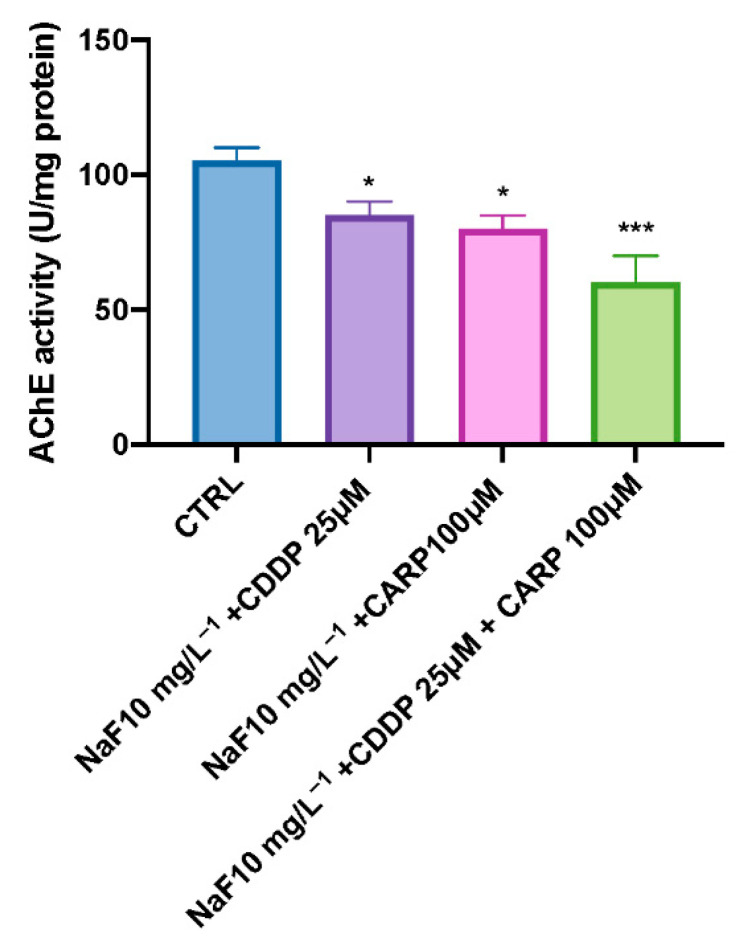
Changes in acetylcholinesterase (AChE) activity in zebrafish larvae after 96 hpf exposure to NaF 10 mg/L + CDDP 25 μM; NaF 10 mg/L−1 + CARP 100 μM; NaF 10 mg/L−1 + CDDP 25 μM + CARP 100 μM Each bar represents 3 replicates (each replicate contained 30 larvae) and expresses as average ± SEM. * *p* ≤ 0.05, *** *p* < 0.001 indicate significant differences between exposure groups and the control group.

As shown in Figure 4, all treatments appear to have a decreased AChE activity, with NaF 10 mg/L + CDDP 25 μM and NaF 10 mg/L^−1^ + CARP 100 μM groups and mostly NaF 10 mg/L^−1^ + CDDP 25 μM + CARP 100 μM group significantly inhibited.

## 4. Discussion

Exposure to xenobiotics, both short and long term, disrupts the redox system, resulting in damage to biomolecules such as DNA, lipids, and proteins. Fluoride, which is found all around the globe in the environment, can form compounds with other elements, causing the CNS to malfunction through a variety of neurotoxic ways. Fluoride can also harm brain tissue by blocking enzymes involved in energy synthesis and transportation, membrane transport, and synapse transport [41]. Recent findings show that pharmaceuticals are evenly dispersed in aquatic ecosystems, necessitating more investigation into their potential effects on aquatic species [42,43]. Pharmaceuticals are different from other pollutants as they are designed to react with specific pathways at low concentrations, which can cause considerable changes in aquatic species. The purpose of this research was to assess the environmental impact and the toxicity of the association of NaF with chemioterapic drugs CDDP and CARP, as well as the possible processes involved in their toxicity pathway. The first step in this study was to evaluate through an embryo toxicity assay, the mortality and hatchability of zebrafish larvae from 24 to 96 hpf. From the results of our previous experiments, we inferred that larva exposed to NaF 50 mg/L^−1^ and 25 mg/L^−1^ showed malformations and developmental toxicity, in contrast to NaF 10 mg/L^−1^ and 5 mg/L^−1^ concentrations that showed no statistically significant signs of developmental toxicity. Following these findings, we decided to use the highest non-toxic concentration of NaF (10 mg/L^−1^), and observe its possible harmful effects in combination with a still non-toxic dose of CDDP (25 μM) and CARP (100 μM). In accordance with the results obtained by the observation of exposed larvae to the three solutions (NaF 10 mg/L^−1^ + CDDP 25 μM; NaF 10 mg/L^−1^ + CARP 100 μM and NaF 10 mg/L^−1^ + CDDP 25 μM + CARP 100 μM), we found an increase in mortality in all groups compared to the control group and even more marked in the NaF 10 mg/L^−1^ + CDDP 25 μM + CARP 100 μM compared to the other exposure groups and mostly versus the control group. Several malformations were found in the treated groups, mostly on the NaF 10 mg/L^−1^ + CDDP 25 μM + CARP 100 μM group, which showed abnormalities in larvae embryogenesis in the 100% larvae of the entire group, and yolk sac edema was the most common type of malformation detected in this group. The larvae hatching rate also showed a clear collapse in all the exposure groups, compared to the control group. In fact, NaF 10 mg/L^−1^ + CDDP 25 μM; NaF 10 mg/L^−1^ + CARP 100 μM and NaF 10 mg/L^−1^ + CDDP 25 μM + CARP 100 μM groups showed no hatched larvae at 72 hpf and a 100% reduction in hatching rate in the NaF 10 mg/L^−1^ + CDDP 25 μM + CARP 100 μM at 96 hpf. Moreover, all treated groups suffer a delay in hatching and an increase in mortality rate versus the control. As hatching is such a crucial step in zebrafish development, structural and functional abnormalities throughout the embryonic stage were ascribed to the reduced hatching rate [44,45]. The difficulties of emerging larvae breaking the eggshell, [46] as well as the inhibition of mitosis or embryogenesis [47], all contributed to the developmental delay. As noted in several previous studies, NaF triggers an inflammatory response and increases oxidative stress on zebrafish animal model [22,48], as well as CDDP [49] and also CARP which produces oxidative stress in other experimental models [50]. Oxidative stress occurs when the steady-state link between the formation of reactive oxygen species (ROS) and the body’s antioxidant defense capacity is disrupted. ROS is continuously produced by active metabolic activities under normal physiological conditions. Tissues have developed a complex array of antioxidant enzymes and free radical scavengers as a defense strategy against ROS [51,52]. Increased lipid peroxidation and DNA breakage in germ cells may be caused by reactive oxygen species (ROS). Antioxidant enzymes are the first line of defense against the harmful effects of reactive oxygen species (ROS) [53]. SOD catalyzes the dismutation of superoxide radicals into hydrogen peroxide (H_2_O_2_) and molecular oxygen, whereas SOD is known to detoxify H_2_O_2_ [54,55]. One of the most common processes resulting from oxidative stress is lipid peroxidation [56,57]. In the current study, we showed that despite individually at the same concentrations do not cause statistically relevant toxic effects [58,59,60], NaF 10 mg/L^−1^ + CDDP 25 μM, NaF 10 mg/L^−1^ + CARP 100 μM, and NaF 10 mg/L^−1^ + CDDP 25 μM + CARP 100 μM treated larvae had considerably higher activity of SOD and CAT, which resulted in dramatically enhanced lipid peroxidation. Furthermore, it has already been observed in other animal models that NaF, CDDP and CARP are also directly involved in cell death mechanisms [22,61,62]. Several studies have largely highlighted the involvement of oxidative stress in the activation of apoptosis processes [63,64]. These correlations between oxidative damage and cell death have also been demonstrated in several studies conducted in both larvae and adult zebrafish [65,66]. We employed a RT-PCR to assess the protein expression levels of larvae exposed to the three solutions (NaF 10 mg/L^−1^ + CDDP 25 μM, NaF 10 mg/L^−1^ + CARP 100 μM and NaF 10 mg/L^−1^ + CDDP 25 μM + CARP 100 μM) at 96 hpf to determine the possible causes of the harmful effects generated by NaF associations with CDDP and CARP. The expression levels of apoptosis-related proteins (caspase-3 and bax) rose in all exposure dose, according to our data, while anti-apoptotic protein bcl-2 expression was significantly inhibited (*p* value < 0.0001). Ultimately, we highlighted a downregulation of AChE activity in the exposure groups versus the control. AChE is a key enzyme of neurotransmitter in biological nerve conduction, and its alteration may be associated with toxic exposure of environmental contaminants [67]. The reduction in activity has been marked especially in the NaF 10 mg/L^−1^ + CDDP 25 μM + CARP 100 μM not only versus the control group but also compared with the other two exposure groups. 

## 5. Conclusions

Finally, NaF and CDDP/CARP co-exposure resulted in altered antioxidant defenses as well as enhanced lipid peroxidation, whereas separate exposures had no significant effects when compared to the control group. In contrast to single concentrations that had no harmful effects, co-exposure of NaF and CDDP or CARP caused not only problems in embryonic development, as well as an imbalance in antioxidant defenses, but also an increase in the apoptotic process. The massive presence of NaF in the environment, and other anthropogenic contaminants, such as platinum-derived anticancer drugs, pose a major health risk to both humans and the various animal species that come into contact with them. Future studies will be needed to elucidate the synergistic toxicity of these contaminants in aquatic species and consequently to humans.

## Figures and Tables

**Table 1:** Primers for real-time PCR.

Gene	Primer Orientation	Nucleotide Sequence
*b-actin*	forward	5′-AGAGCTATGAGCTGCCTGACG-3′
	reverse	5′-CCGCAAGATTCCATACCCA-3′
*casp-3*	forward	5′-CCGCTGCCCATCACTA-3′
	reverse	5′-ATCCTTTCACGACCATCT-3′
*Bax*	forward	5′-GGCTATTTCAACCAGGGTTCC-3′
	reverse	5′-TGCGAATCACCAATGCTGT-3′
*bcl-2*	forward	5′-TCACTCGTTCAGACCCTCAT-3′
	reverse	5′-ACGCTTTCCACGCACAT-3′

**Table 2:** NaF, CDDP, and CARP effects on zebrafish larvae at 96 hpf.

	Malformations	Mortality				Hatching			
	96 h	24 h	48 h	72 h	96 h	24 h	48 h	72 h	96 h
CTRL	0	0	0	0	0	0	8%	96%	100%
NaF									
50 mg/L^−1^	SC, PE and YE	0	0	82%	100%	0	0	100%	100%
25 mg/L^−1^	SC	0	0	11%	15%	0	0	15%	85%
10 mg/L^−1^	0	0	0	4%	5%	0	0	90%	95%
5 mg/L^−1^	0	0	0	0	0	0	3%	95%	100%
CDDP									
50μM	SC PE and YE	0	0	30%	60%	0	0	0	0
25 μM	0	0	0	0	0	0	0	0	100%
10μM	0	0	0	0	0	0	0	0	100%
5 μM	0	0	0	0	0	0	6%	94%	100%
CARP									
150μM	SC, PE and YE	0	0	40%	75%	0	0	75%	0
100μM	0	0	0	0	0	0	0	0	100%
50μM	0	0	0	0	0	0	0	0	100%
25 μM	0	0	0	0	0	0	5%	20%	100%

Scoliosis (SC); Pericardial edema (PE); Yolk sac edema (YE).

## Data Availability

Not applicable.
